# Account of Several Cases of Carditis: With Remarks

**Published:** 1835-01-01

**Authors:** William Stroud

**Affiliations:** Physician to the Northern Dispensary, &c.


*Account of several Cases of Carditis: with Remarks.* Communicated to the Harveian Society, by William Stroud, m.d. Physician to the Northern Dispensary, &c.

(Continued from page 198.)

Before proceeding to describe the two remaining cases of carditis, formerly promised, it may not be unacceptable to adduce another example of the same kind, serving, like that last related, to illustrate the intimate connexion which often subsists between the diseases of the heart and those of the brain.

The influence exercised by the heart on other organs is subject to considerable variety with respect to its mode, duration, and degree, as well as to the medium by which it is transmitted. In some instances, the effect is produced by an increased supply, or an impeded return of blood; in others, perhaps, by the impaired, or vitiated quality of the blood. Sometimes it is the result of functional disorder of the cardiac nerves; and, at other times, of structural disease, extending along the vascular trunks to some of their remote branches. To the class last mentioned may be referred the case now about to be detailed, in which a morbid state of the arteries of the brain, attended with temporary derangement of mind, was induced by chronic inflammation of the heart and its appendages, with serous effusion into the pericardial sac. Under such agency, and partly, perhaps, through the influence of certain obscure causes of metastasis more immediately connected with vital action, a long course of debility and depression was followed by a transient, but severe, paroxysm of maniacal excitement, which remarkably modified the aspect of the complaint, and accelerated its fatal termination.

October 17, 1830.—I was desired to visit, as a patient of the Northern Dispensary, Maurice H****e, a fine looking man, about seventy years of age, and more than six feet in height, who had formerly served for nearly thirty years in the Life Guards, but had never been in action. During the earlier part of his life he enjoyed good health; but, latterly, had been for several years in a declining state; which, independently of his advanced age, might in some measure be ascribed to intemperate habits, and to the care and anxiety attendant on impoverished circumstances. He was a man of placid temper, but of an active and enterprising disposition, fond of trying speculations, which generally contributed to impair his fortune, and of taking medicines of his own prescription, which probably tended to injure his health. He had been three times married, and had granddaughters, who attended him in his last illness. His third marriage, which was never consummated, took place within a year of his death.

I found that he had been labouring for two years under a pectoral complaint, bearing the general aspect of Syncope Anginosa, liable to aggravations, more especially in winter; and for which, about a year since, he had been bled with some relief. His principal present symptoms are attacks of spasmodic action in the heart, occurring during the night, or very early in the morning, attended with a distressing sensation like that of drowning, by which he is roused from sleep with alarm, and compelled to sit up in bed. He has no sympathetic pain in the arms or abdomen; and, with the exception of being weak and low-spirited, has little other complaint. About twenty-five years since, he suffered from acute rheumatism, chiefly affecting the arms, but in which the thoracic organs did not seem to participate.

*Prescriptions.* Sumantur primo mane, prout opus fuerit, Pil. Rhei comp. gr. v. vel. x.; et Extract. Hyoscyam. gr. v. omni vespere.—Aquæ puræ f℥vj; Potass. Acetat. ʒivss.; Tinct. Digital. fʒss.; Tinct. Zingiber. fʒij. Sumatur f℥j. ter in dies.

27. Without taking the composing pill, he sleeps rather better, but is sometimes disturbed by frightful dreams, concerning water, and drowning. His urine is now extremely copious, almost amounting to diabetes, especially during the night; and its quantity is always observed to be in an inverse proportion to the intensity of the pectoral symptoms, being deficient when they are severe, and abundant when they are moderate. He has lately had a spasmodic pain in the calf of the left leg.

*Prescriptions.* Aquæ puræ f℥ivss.; Mucilag. Tragacanth. f℥iss.; Pulv. Zingiber. ℈j.; Pulv. Rhei ʒss.; Magnes. Calcinat. ʒj.; Tinct. Hyoscyam. fʒj.; Tinct. Cardamom. comp. fʒij. Sumatur f℥j. ter in dies.

November 11. The complaint is gradually increasing, with general debility, and depression. The pulse is irregular, and intermitting, and the respiration is frequently accompanied with a slight groan. The urine is less copious. During the night he is frequently compelled to sit up in bed, with his body inclining forwards; but, in the daytime, he can lie down, and sleep with comparative ease.

*Prescriptions.* Extrah$^{r}$ per. V. S. sang. f℥viii. vel. xij. Elaterii gr. vj.; Pulv. Zingiber. ℈ij.; Syrupi q.s. Fiant Pil. xij., quarum sumatur una, omni horâ, donec alvus soluta fuerit. Sumantur omni vespere Pil. Sapon. cum Opio. gr. v. vel. x. Per intervalla Rep$^{r}$. Potio Rhei cum Magnesia.

26. The complaint seems progressively to increase, the more so, perhaps, in consequence of the patient being capricious, and irregular in the use of medicines. In addition to his other complaints, he has for some time had a cough, with thick yellow expectoration, and an uneasy sensation of spasm, and suffocation, at the pit of the stomach, which is relieved by eructation. He is weak, low-spirited, and restless; groans, or mutters much to himself, lies down with difficulty, sleeps ill, and often wakes in a fright, more especially about two or three hours after midnight. This symptom was at the time relieved by the bleeding. His bowels are regular, and his urine is less copious than formerly. On examination with the stethoscope, the action of the heart is found to be very feeble and obscure, while the respiratory murmur is loud and puerile.

*Prescriptions.* Pectori medio admovr. Emplastr. Cantharid. —Extract. Hyoscyam. ℈j.; Pulv. Scillæ, Pulv. Digital., Pulv. Cambog. *aa* gr. vj.; Syrupi q. s. Fiant Pil. xij., quarum sumatur una ter in dies.—Mist. Camphor. f ℥vi.; Tinct. Scillæ f ʒj.; Tinct. Hyoscyam. f ʒij.; Spir. Æther. Nit. f ʒiij.; Sumatur f ℥ j. omni vesp. horâ somni, et Repr. media nocte p. o. f.

December 1. The blister operated well, and the discharge produced by it still continues. The bowels are relaxed. The dejections are watery, and fetid. The urine is again copious, yet the ancles are a little œdematous. The cough is occasionally troublesome, with much mucous expectoration. He sleeps little, either by night or by day, but is lately observed to be somewhat stronger, and his appetite is increased.

*Prescriptions.* Rep. Med.

18. During the last week the symptoms of delirium tremens have gradually supervened, accompanied with much heat, and perspiration of the head, and an almost total interruption of sleep. A few days since, the action of the heart was examined by the stethoscope, and found to be stronger than before. The bowels continue to be rather lax.

*Prescriptions.* Infus. Sennæ f ℥ivss; Aquæ Menth. vir. f ℥iss.; Magnes. Sulphat. ℥iss.; Sumatur f ℥ j., primo mane, p. o. f. Pulv. Glycyrrhiz. Sulphat. Quinin. *aa* gr. xij.; Pulv. Opii gr. vj.; Syrupi q. s. Fiant Pil. xij., quarum sumatur una ter, vel quater in dies.

24. The patient soon afterwards became furiously maniacal; and, after having long been weak, helpless, and dispirited, was now strong, active, and dangerous. His wife and granddaughters being unable to control him, and dreading ill consequences to himself or others, if he were left at liberty, procured his removal this day to a neighbouring asylum, where he remained till his death, which took place five weeks afterwards. In his new dwelling, a blister was applied to the crown of his head with some relief; and, by the aid of mental management, and proper diet, the cerebral symptoms after a time abated, while the pectoral complaints were renewed, particularly shortness of breath on attempting to ascend a stair, a disposition to sit with the body leaning forwards, as if to relieve an uneasy sensation about the heart, and some œdema of the legs. After this, his strength rapidly declined, and he at length died rather suddenly, on the morning of January 31, 1831.

*Post-mortem Appearances.* The body, having been conveyed to the patient's former lodging, was inspected by me, on the 2d of February, when the following appearances were observed.

*General Conditions.* The body was tall, well made, and not emaciated ; but, with the exception of the heart, and great vessels, contained little blood, and no fat. The skin was moderately firm ; the muscles were soft and flabby; the cellular substance of the abdomen was loose, watery, and verging to putrescence. The limbs were plump, and the lower extremities were somewhat œdematous.

*Head.* The crown, which was bald, exhibited several red, and dry spots, probably occasioned by blistering. The scalp adhered to the calvaria by a thin layer of very dense cellular membrane, which rendered it difficult to denude the bone. The dura mater was thick, and white. It adhered in a few places to the inner table by long slender filaments, as likewise to the subjacent membranes, on each side of the longitudinal sinus at the vertex. The arachnoid covering the hemispheres was somewhat thickened, and œdematous; and the pia mater was much injected. On dividing the dura mater, a good deal of reddish serum escaped from between the membranes. The substance of the brain was firmer than usual, more especially the medullary portion, which, on section, exhibited several bloody points, but was otherwise sound. The cerebellum was softer than the cerebrum, but the component parts of the medulla oblongata were very distinctly marked. The encephalic nerves were rather small, as if wasted. The arteries were thick and rigid, with some tendency in the basilar artery to cartilaginous or osseous degeneration. Owing to their patulous state, much liquid blood escaped from the divided carotid, and vertebral arteries, when the atmospheric pressure was admitted, by opening the chest. There was a little liquid in the ventricles, chiefly on the left side. The choroid plexuses were rather congested, but not hydatiform. The commissures were very dense, and firm.

*Thorax.* The cartilages of the ribs were ossified, requiring the use of the saw for their division. The second pair of ribs was extuberant. The mediastinum was thick, and fleshy. The pleural sacs contained a large quantity of bloody serum. The membrane was not inflamed, but its pulmonary reflexion was slightly attached to the diaphragm and sides by a few slender adhesions. The lungs were voluminous, and emphysematous, but otherwise healthy. The decumbent portions were a little congested. The external surface was beautifully mottled in the manner frequently observed in old persons, with dark purple spots, about the size of a pea, regularly sprinkled on a light red ground. On making sections into the lungs, there was found little or no effusion, either bronchial, or parenchymatous ; but the lining membrane of the trachea was very red, and the posterior band of longitudinal fibres was remarkably distinct. The pericardium was white, and rather thick, presenting a blanched or sodden appearance, like that of the peritoneum in ascites. It contained several ounces of pale, watery liquid; and, by its loose, and flaccid condition, seemed to have formerly contained a greater quantity. The heart was large, more especially before its contents, and those of the pericardial sac, had been removed. The auricles were dilated, and inclosed much liquid blood; but there were few coagula except in the ventricles. The latter were considerably hypertrophied, and very firm, particularly the left ventricle. The valves, both cardiac, and arterial, were perfect. The aorta and the pulmonary artery were large, and rather thin. The orifices of the coronary arteries were patulous, and included between them a sharp ridge, or crest of ossification, no where else observed.

*Abdomen.* The peritoneal sac contained a little pale serum. A considerable portion of the jejunum, and of the ileum was red contracted, and somewhat twisted, or retroverted. The liver was of full size, but healthy; and the gall-bladder was distended by thin bile. The spleen was short, and quadrangular; and, like the liver and kidneys, was firm in substance, uniform in texture, and of a deep red colour. The convex edge of the right kidney was indented with depressions resembling cicatrices, giving the organ a lobulated appearance. The bladder was contracted, and empty.

Remarks. The general character of the disease just described was chronic inflammation of the heart, and ultimately of the brain, attended with serous effusion, and other organic lesions. Its apparent causes were habitual, but not excessive intemperance; grief, and anxiety on account of pecuniary losses; and perhaps, also, acute rheumatism in the arms, which occurred some years before, but of which the influence was more doubtful. The two former causes might have operated at once on the heart, and on the brain, and each of these organs may have affected the other; but, as the cardiac symptoms existed alone for nearly two years, while the cerebral ones did not supervene till within the last two months, the latter, which, perhaps, were chiefly concerned in accelerating the fatal termination, must be regarded as secondary to the former. In both organs, the inflammatory action, although destructive, was moderate, and the physical results corresponded.

In the pericardium, as in the peritoneum, and other serous membranes, it is evinced by the blanched, or sodden aspect produced by thickening, and opacity, depending, no doubt, on an interstitial deposit of solid albumen, which, in a liquid form, is at the same time discharged in excess from the secreting surface. The loose, and flaccid state of the pericardial sac, notwithstanding the heart itself was enlarged, seemed clearly to indicate that it had once contained a greater quantity of liquid than was actually found in it. This, of course, implies the possibility of absorption taking place from that cavity under favourable circumstances, including, perhaps, a low degree of inflammatory action in the membrane itself, and an abundant flow of urine, which, by its sympathetic influence on the kidneys, it seems occasionally, as in this case, to excite. The excessive growth and firmness of the fleshy substance of the ventricles, and the incipient ossification between the orifices of the coronary arteries, were the natural consequences of the same inflammatory action, not confined to any one texture, but gradually pervading every portion of the organ attacked. The dilated state of the auricles, and the copious extravasation of bloody serum into the pleural sacs, might reasonably be ascribed to languid action of the ventricles, and consequent obstruction of the circulation at its centre, conjoined with relaxation of the capillaries, immediately preceding death; and it was, probably, owing to the same defect of vital action that the detained blood was nearly liquid.

The existence of chronic inflammation in the brain, and its appendages, was denoted by close adhesion of the scalp to the cranium, thickening, or injection of the cerebral membranes, effusion of reddish serum between them, cartilaginous rigidity of the arteries, and increased firmness of the medullary substance, with a display of bloody points on section. Either of these conditions separately, and especially the whole of them combined, is abundantly sufficient to derange the functions of the brain, and, in some cases, to destroy life. The other morbid states of this organ might in a great measure have been derived from that of the cerebral arteries, and originally from that of the heart, not by continuous sympathy, since the great vascular trunks were unaffected, but through the medium of the ganglionic nerves, which enter into the composition of blood-vessels, as of all other parts, and exercise an important influence, both on their nutrition, and on their actions. Such a state of the arteries strongly disposes to disease of the brain, and of its membranes, yet it sometimes exists alone; and, tending, at length, by the brittleness which it occasions, to sudden rupture from slight causes, produces a distinct variety of apoplexy, exclusively vascular.

The traces of inflammatory action in the abdomen were inconsiderable; but it is not improbable that a long course of moderate intemperance, conjoined with mental vexation, in a person of good constitution, may give rise to that increased firmness, and redness of all the solid viscera of this region, which was here observed, and which may justly be regarded as the product of chronic irritation, and the prelude to organic disease.

After duly considering the history and pathology of this case, its symptoms are easily explained. When the brain is not specially predisposed, cardiac disorder may long continue without exciting much cerebral disturbance, although it is usually productive of susceptible feelings, and of an irascible temper. In this instance, habitual depression of mind, approaching to despondency, constituting the lower degree of mental derangement, ultimately advanced to the higher, and more conspicuous degree, marked by delirium tremens, constant watchfulness, and maniacal excitement. On the other hand, cerebral disorder generally affects the heart, producing, when acute, and unattended with effusion, a strong and hard pulse, but more commonly, a weak, and very frequent one, and, in some cases, intermission, and a tendency to syncope. Such might in this case have been, in part at least, the cause of the feeble and obscure action of the heart which long prevailed, but which might partly, also, be ascribed to the mechanical obstruction occasioned by an accumulation of liquid in the pericardial sac, and partly, perhaps, to that debilitating influence which certain irritations of membranous parts frequently exercise on organs with which they are associated, producing torpor and relaxation, and, ultimately, extenuation and atrophy. The law of vital action on which this influence depends, is one of great importance, and demands a fuller development than can here be attempted; but examples of its agency are occasionally furnished by scarlatina, cholera, inflammation of the bronchia, and of the peritoneum, and by various forms of intestinal irritation. To the same agency may, perhaps, be attributed the inflated, or emphysematous state of the lungs here observed, a state for which mere mechanical distention, although it may no doubt partially contribute, is unable fully to account.

The greater interruption of sleep in the night than during the day is a circumstance meriting attention; depending, probably, on cerebral influence, rendering the sleep in the first place more complete, so as to occasion a temporary suspension of the heart's action, already feeble and embarrassed, which is naturally followed by uneasy sensations, and by a violent, and convulsive effort to restore the circulation. Hence, persons labouring under such complaints may sometimes be prevented from waking in alarm, by their attendants watching the first approach of restlessness, and dyspnœa, and gently tapping them on the chest, or back, so as, without actually rousing them, to render their sleep for a time less profound.

The influence of the diseased heart on the organs situated in the upper region of the abdomen is often very considerable, and seems in a great measure to depend on its physical qualities of increased weight, heat, and impulse, freely operating through the thin partition of the diaphragm, and giving rise to various forms of gastric, or hepatic irritation. In cases of hypertrophy with strong palpitation, pain in the epigastrium, with nausea and vomiting, is not an unfrequent occurrence; and, independently of intemperance, the liver is often enlarged, and mottled, so as to present on section an aspect like that of nutmeg, owing, as it appears, to venous congestion occasioned by the difficult return of its blood. The stomach, in its turn, acts powerfully on the heart. Hence the ill effects arising in cardiac complaints from strong emetics, active stimulants, a full meal, especially in the evening, flatulent, or indigestible food, and various other errors in diet; whilst, on the contrary, the seasonable application to the epigastrium of leeches, blisters, and opiate liniments, and the moderate use of calomel and rhubarb, as a cholagogue purgative, are often productive of much benefit.

The occasional redundance of urine in such cases is calculated to give relief, not merely as a mode of depletion, but also, by the sympathetic influence of the kidneys on the pericardium, agreeably to the law of vital action, whereby all secreting surfaces throughout the system mutually affect each other, the increase of the renal function being at once a sign, and a cause of that incipient reduction of the pericardial inflammation, of which, when moderate, it is a natural result.

The diagnosis in this case was facilitated by the absence of any material complication, and by the harmonious concurrence of the general symptoms, the physical signs, and the previous history of the disease, in furnishing the same indication of its nature. The distressing sense of spasmodic action in the præcordia, the terrifying dreams concerning water, and drowning, the sudden disturbance of sleep in the early part of the morning, the necessity of sitting up in bed, and the relief obtained by inclining the body forwards, together with the weak, and intermitting pulse, and the dull, and indistinct sound of the heart's action, were all significant of the same state of cardiac disease, which intemperance, anxiety, vexation, and acute rheumatism were most likely to have produced. Owing to the unavoidable hurry and imperfection of Dispensary practice, especially when persons are to be visited at their own dwellings, the physical signs derivable from auscultation and percussion were not sufficiently explored; but, as far as they were consulted, their information was valuable, and fully confirmed by the result. In consequence of the diversity and mutability of circumstances, these signs are, however, often of a various, and sometimes of an opposite character. In chronic pericarditis, with serous effusion, the action of the heart is generally very feeble and obscure, although it may be diffused over a wide space, and the arterial pulse may at the same time be occasionally full, and strong. Pericarditis with universal adhesion is, on the other hand, frequently attended with violent palpitation, contrary to the opinion of Allan Burns, and others, who, on speculative grounds, have regarded the two conditions as incompatible. In this instance, the increased action of the heart, following the copious discharge of urine, and immediately preceding the stage of cerebral excitement, was very observable; and, had it been properly appreciated at the time, might, perhaps, have prompted measures for the prevention, or mitigation of that dangerous state.

The prognosis of complaints of this kind must, of course, be generally unfavourable, chiefly on account of the irreparable organic changes which have usually taken place before medical aid is requested. In consideration of their inflammatory character, although chronic, and attended with delusive symptoms of depression, the general plan of treatment should be moderately antiphlogistic. Active depletion is for the most part inadmissible; since, without curing the disease, it rapidly exhausts the strength of the patient; but all the remedies employed, however strictly restrained within due limits, should be selected from the debilitating class. Small occasional bleedings, the application of leeches, blisters, and narcotics to the epigastrium, or of a seton between the shoulders, combined with spare diet, tranquillity of mind and body, the diligent promotion of the several excretions, especially that of urine, and the moderate use of sedative and revulsive medicines, such as tartarized antimony, nitre, digitalis, colchicum, and hyoscyamus, are the means which reason and experience unite to suggest as best adapted to relieve the urgent symptoms of such complaints, to retard their progress, and to repair, as far as possible, the injury which they may already have committed.

[The remaining cases of carditis will be inserted in the ensuing number.—EDITOR.]

